# Innovative non-thermal plasma disinfection process inside sealed bags: Assessment of bactericidal and sporicidal effectiveness in regard to current sterilization norms

**DOI:** 10.1371/journal.pone.0180183

**Published:** 2017-06-29

**Authors:** Zouhaier Ben Belgacem, Gaëlle Carré, Emilie Charpentier, Florian Le-Bras, Thomas Maho, Eric Robert, Jean-Michel Pouvesle, Franck Polidor, Sophie C. Gangloff, Mohamed Boudifa, Marie-Paule Gelle

**Affiliations:** 1 Laboratoire de Biomatériaux et Inflammation en Site Osseux (EA 4691), SFR CAP-Santé, Université de Reims Champagne-Ardenne, Reims, France; 2 CRITT-MDTS, Charleville-Mézières, France; 3 Groupe de Recherches sur l’Energétique des Milieux Ionisés (UMR 7344), Orléans, France; 4 Sominex, Bayeux, France; Universite Toulouse III Paul Sabatier, FRANCE

## Abstract

In this work, we developed a device capable to generate a non-thermal plasma discharge inside a sealed bag. The aim of this study was to assess the effectiveness of the oxygen, nitrogen and argon plasma sterilization on *Pseudomonas aeruginosa*, *Staphylococcus aureus* and *Bacillus subtilis* spores according to the NF EN 556 Norm. Moreover the bag integrity which is a critical key to maintain the sterile state of items after the end of the process was verified by Fourier Transform Infrared (FTIR) and X-ray Photoelectron Spectrometry (XPS) analyses. After plasma treatments, the bacterial counting showed a 6 log reduction of *P*. *aeruginosa* and *S*. *aureus* in 45 min and 120 min respectively whatever the gas used and a 4 log reduction of *B*. *subtilis* spores in 120 min with only oxygen plasma. These results were confirmed by Scanning Electron Microscopy (SEM) observations showing altered bacteria or spores and numerous debris. Taking into account the studied microorganisms, the oxygen plasma treatment showed the highest efficiency. FTIR and XPS analyses showed that this treatment induced no significant modification of the bags. To conclude this non-thermal plasma sterilization technique could be an opportunity to sterilize heat and chemical-sensitive medical devices and to preserve their sterile state after the end of the process.

## Introduction

The sterilization of medical devices has always been a very important issue in hospitals so as to prevent nosocomial infections. Sterilization is defined as a complete inactivation of any forms of life and more precisely as the definitive inability of microorganisms to replicate. Sterilization is nowadays most commonly achieved by high pressure saturated steam (autoclaving, EN 554), ethylene oxide (EtO) treatment (ISO 11135 / EN 550), or by ionizing radiation (EN 552). High pressure saturated steam leads to the destruction of microorganisms’ key molecules and structures such as DNA, RNA, proteins, and lipids. At the same time, this technique can severely damage thermosensitive polymer medical devices by affecting their physical and mechanical properties and then potentially their biocompatibility [1]. Though chemical sterilization such as EtO is a relatively low temperature process (65°C maximum), it features some drawbacks: long processing times and long ventilation periods before being able to use sterilized items [2]. Some authors [3–4] have moreover raised questions about the possible carcinogenic or failure fertilisation properties of the EtO residues adsorbed onto the items after processing. The gamma radiation process is conducted close to ambient temperature and leads to DNA lesions of microorganisms. This technique could however affect some properties of the treated polymers by breaking some of their bonds and cross-linked chains. This process is also costly and requires high-level safety equipment [5].

Nowadays, numerous new materials are being developed but they are not compatible with the above mentioned standard sterilization treatments [1, 6]. These various methods’ limitations require the development of new alternative techniques to provide: 1) the possibility or ability to preserve the sterile state of medical devices after the end of the sterilization process; 2) a processing temperature which does not exceed 40°C a temperature lower than for temperature of EtO treatment (65°C); 3) the possibility to treat a wide range of medical devices without modifications of their mechanical properties and their biocompatibility; 4) a process which must be harmless for operators and patients; 5) an ecological process which requires less energy and reduces running costs.

The use of non-thermal plasma has been studied as an alternative standard sterilization process since the end of the sixties [7]. Plasma is an ionized gas consisting of ions, electrons, ultra-violet (UV) photons, neutral species and reactive oxygen and nitrogen species (RONS) with sufficient energy to break covalent bonds and to initiate various chemical reactions [8]. Numerous techniques using non-thermal plasma have been developed over the last few decades [7, 9–12], and have demonstrated the effectiveness of plasma treatment on the inactivation of various bacterial strains such as *Bacillus subtilis* (*B*. *subtilis*) [13–15], *Pseudomonas aeruginosa* (*P*. *aeruginosa*) [16, 17], *Staphylococcus aureus* (*S*. *aureus*) [18,19], *Staphylococcus epidermidis* [19]. However, these techniques that produce non-thermal plasma differ from each other in terms of electrical current, discharge reactor designs, operating pressures, and operating conditions (nature and flow rate of the gas). This explains why it is so difficult to compare the overall results. Crucially, these techniques could only provide sterilized objects up to the end of the process and not afterwards. It is due to the lack of preconditioning (pre-packaging) items which is a capital step to ensure their sterile state during transportation and storage before their use [8, 20–21].

According to Moisan *et al*. [6], the sterilization of pre-packaged materials is the successful solution to preserve the sterile state, although this step seems to be very problematic. To overcome this issue, they propose two solutions which are difficult to apply: 1) to apply the plasma through the previously sealed package resulting in potential loss of effectiveness of some ionized species interacting with the bag membrane. Sarrette *et al*. showed that in some plasma conditions N-atoms were able to go through polypropylene membranes without any loss [22]; 2) to mechanically pack the item directly in the vacuum chamber at the end of the plasma treatment (this solution seems extremely difficult to implement under low pressure conditions). However, sterilization treatment directly carried out in a sealed bag required to solve methodological issues [23, 24].

The aim of the present study was to evaluate the sterilization effectiveness of our equipment (patent WO 2012/038669 A1) performing a non-thermal plasma discharge inside a sealed bag at low pressure on *P*. *aeruginosa* or *S*. *aureus* and *B*. *subtilis* spores, used as sterilization bioindicators in the norm NF EN 556. Oxygen, nitrogen and argon plasma were tested on these microorganisms. This non-thermal plasma equipment is intended to sterilize single use medical devices which are resistant to low pressure. The bag integrity, which is a critical key to maintain the sterile state of items, was verified by FTIR and XPS analyses downstream the non-thermal plasma process.

## Material and methods

### Bacterial strains

*Pseudomonas aeruginosa* CIP 82.118 (Gram-negative bacteria) and *Staphylococcus aureus* CIP 53.154 (Gram-positive bacteria) were provided from Pasteur Institute (Paris, France). Bacteria were stored at −80°C in an appropriate medium supplemented with 50% glycerol. Bacteria were cultured in a specific nutrient medium made from D(C) glucose, peptone from casein and soybean and sodium chloride. Three sub-cultures were made and then incubated at 37°C in a rotary shaker at a constant speed (250 g) to obtain a final bacterial concentration of 10^8^ Colony Formant Unit (CFU)/mL. For sterilization studies, bacteria were centrifuged at 4,200 g for 10 min at 4°C and washed twice with buffered peptone water at pH 7. The resulting cellular suspension was adjusted to 10^8^ CFU/mL and 20 μwere deposited on sterilized glass slides (15 mm x 15 mm) and then dried at 37°C for 10 min before plasma treatment.

### Bacterial spores

*Bacillus subtilis subsp*. *spizizenii* ATCC 6633 provided from Mesalabs was used as biological indicators to determine the efficiency of the process. The spores were stored at −20°C in 40% ethanol solution. To prepare the spore suspension, they were diluted in distilled water to obtain a suspension at 2.6.10^7^ spores/mL and 20 °L were deposited on sterilized glass slides (15 mm x 15 mm) then dried at 37°C for 15 min before plasma treatment under microbiological safety hood.

### Plasma generation in sealed sterilization bag

**Fig 1:**
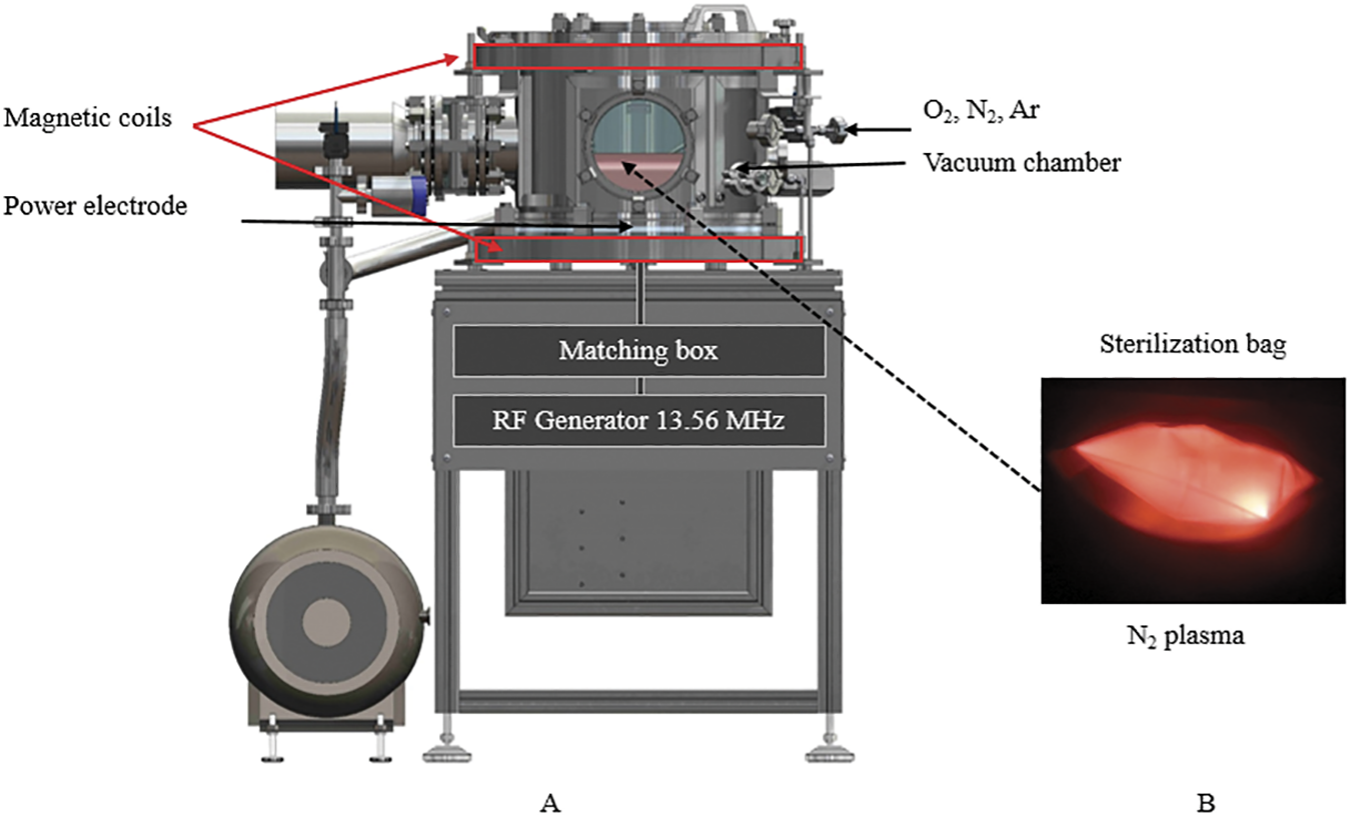
Plasma sterilization prototype device and creation of plasma inside sealed bags. (A) The vacuum chamber was equipped with 2 pumps, a radio frequency polarization plate and two magnetic coils. (B) Creation of N_2_- gas plasma inside a sealed bags.

The prototype manufactured by Sominex (Bayeux, France) consisted of a stainless steel vacuum chamber (35 L) allowing to treat voluminous medical devices (Fig 1A) (Patent Popot and Gelle, 2012) [25]. A high vacuum state was obtained by using two pumps (Agilent TRISCROLL 300 and Agilent V301). The plasma discharge was generated by a radio-frequency (RF) polarisation plate (Ø 300mm; RF generator: 13.56 MHz; 300 W) coupled with two magnetic coils (0–14 Gauss; SEF, Labege; France) located at the top and the bottom of the vacuum chamber. Three mass flow controllers were connected to the gas lines (O_2_, N_2_ and Ar) to control the flow rate from 0.5 to 1 sccm.

The sterilization bags with a wall thicknesses of 125 °m, made from a low density polyethylene with two Tyvek^®^ porous membranes, were provided by SüdPack^®^ Medica (Germany). These bags are already on the market for the sterilization process and are in accordance with the NF EN ISO 11607, EN 868, NF EN 868–3 and EN 868–6 Norms.

Four glass slides contaminated by bacteria or spores were placed in the bag that was sealed and then set on the RF polarisation plate. When the vacuum reached 1.45 10^−4^ mbar, the gases were injected through the first Tyvek® membrane and the excess gas was released through the second Tyvek® membrane into the vacuum chamber. Then the discharge was induced by the RF polarization (25W to 100W) inside the bag and the plasma was enhanced by the magnetic fields (14 Gauss). Controlling the pressure difference between the vacuum chamber and the bag, the plasma is kept confined inside the bag (Fig 1B) [26].

The temperature inside the bag was checked by surface temperature indicating strips (Thermographique®; Fisher Scientific). At the end of the process, the pumping system was stopped and nitrogen was injected into the vacuum chamber until the system returned to atmospheric pressure. After treatment, the residual live bacteria and spores were counted.

### Plasma effectiveness on *P*. *aeruginosa* and *S*. *aureus*

Contaminated glass slides were exposed either to O_2_, N_2_ or Ar plasma treatments for varying exposure times of 5, 15, 30, 45, 60 and 120 min. For each gas plasma treatment and each time period, at least six independent experimentations were performed in triplicate. The controls consisted of non-exposed samples to plasma as well as samples exposed only to low pressure and not to the ionized gas. Bacteria were collected from glass slides by mechanical agitation in buffered peptone water. These soft sonication and vortex procedures were previously demonstrated to be without effect on bacteria viability [27]. Both bacterial dilution and seeding on nutrient agar plates were performed with EasySpiral Pro® (Interscience, France). After a 24h-incubation at 37°C, the colonies on agar plates were counted automatically with GammeScan®1200 (Interscience, France).

### Plasma effectiveness on *B*. *subtilis* spores

Contaminated glass slides were exposed to O_2_, N_2_ or Ar plasma treatments for varying exposure times of 5, 15, 60 and 120 min. Untreated controls were also performed for each independent experimentations. Six independent experimentations were performed in triplicate. Spores were also collected from glass slides by mechanical agitation distillate water. To be able to numerate spores still alive, the collected spores were heat activated at 80°C for 15 min and then cooled in an ice bath for 10 min to induce their germination. Bacterial suspensions were filtered on cellulose acetate membrane filter (pore size 0.2 μzm, diameter 25 mm, Sartorius, Germany). The filters were then placed in a nutrient agar plates. Spore counts were determined by cultivation on cellulose acetate filter deposited on nutrient agar plates at 30°C. Thus each CFU represents one surviving spore which has been able to germinate, outgrowth and multiply to form a visible colony.

### Scanning electron microscopy (SEM)

SEM observations of plasma treated contaminated glass were set to visualized the morphological changes of bacteria and spores. Contaminated glass slides were fixed with 2.5% glutaraldehyde in phosphate buffer solution (PBS) for 1h at room temperature, and rinsed two times with PBS for 10 min. The samples were dehydrated in graded series of ethanol/water solutions at 50%, 70%, 90% and 100% (twice) then covered with hexamethyldisilazane solution (Sigma-Aldrich^®^) [28]. The samples were desiccated overnight at room temperature then placed at 37°C.

The samples underwent a gold sputtering process using a JEOL JFC 1100 ion sputter. Bacteria were then examined with a scanning electron microscope JEOL JSM 5400 Low Vacuum and the spore samples were observed on a FEG Scanning Electron Microscope Zeiss Ultra Plus because of their extremely small size.

### Plasma bags analysis

For the purpose of the effect of the plasma on the treatment bags, two different analysis level have been carried out. First, Fourier Transform Infrared (FTIR) measurements were collected to determine if a chemical reaction of the plasma exposed bags had taken place. Chemical changes produced at the surface after plasma treatment were studied using FTIR with Attenuated Total Reflectance (ATR) accessory. FTIR spectra were recorded with Perkin–Elmer System 2000, in the 4000–500 cm^-1^ wave-number range with 10 scans at a resolution of 2 cm^-1^. Second, X-ray Photoelectron Spectrometry (XPS) analysis was utilized to determine the changes of the chemical composition at the internal surfaces of the sealed bag using the ESCALAB 250 system (Thermo-VG Scientific Co., Ltd., England). The X-ray source was Al Kα (15 kV) at an operating power of 150 W and a 500 μm in spot size.

### Statistical analysis

The bactericidal effect (BE) or sporicidal effect (SE) were determined by log N_0_—log N_t_, where N_0_ and N_t_ are the number of CFU/glass slide or spores/glass slide in control and treated samples respectively. The data are presented as the mean ± standard error of the mean of at least six independent experimentations performed in triplicate. Statistical analysis was performed by the Student's t test (Prism 5, GraphPad Software). A value of p < 0.05 (*), p<0.01 (**) was considered to be statistically significant.

## Results

### Effectiveness of plasma treatment on bacteria

**Fig 2:**
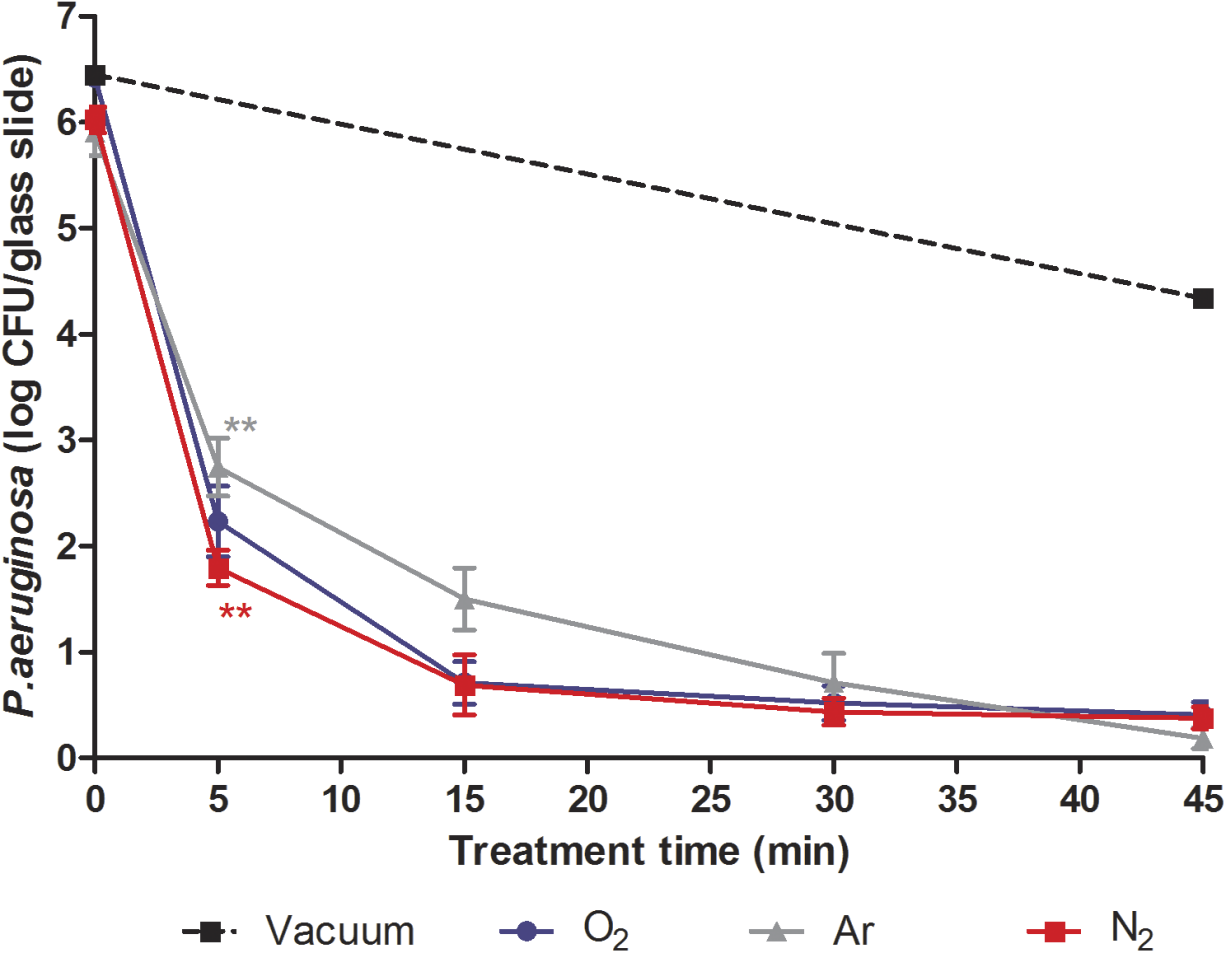
Survival curves of *P*. *aeruginosa* exposed to N_2_, O_2_, Ar non-thermal plasma (25 W; 14 G and 0.5 sccm). Each data represents the mean of data obtained from at least six independent experiments made in triplicate. Statistical analysis was performed by the Student's t test (**p<0.01).

**Fig 3:**
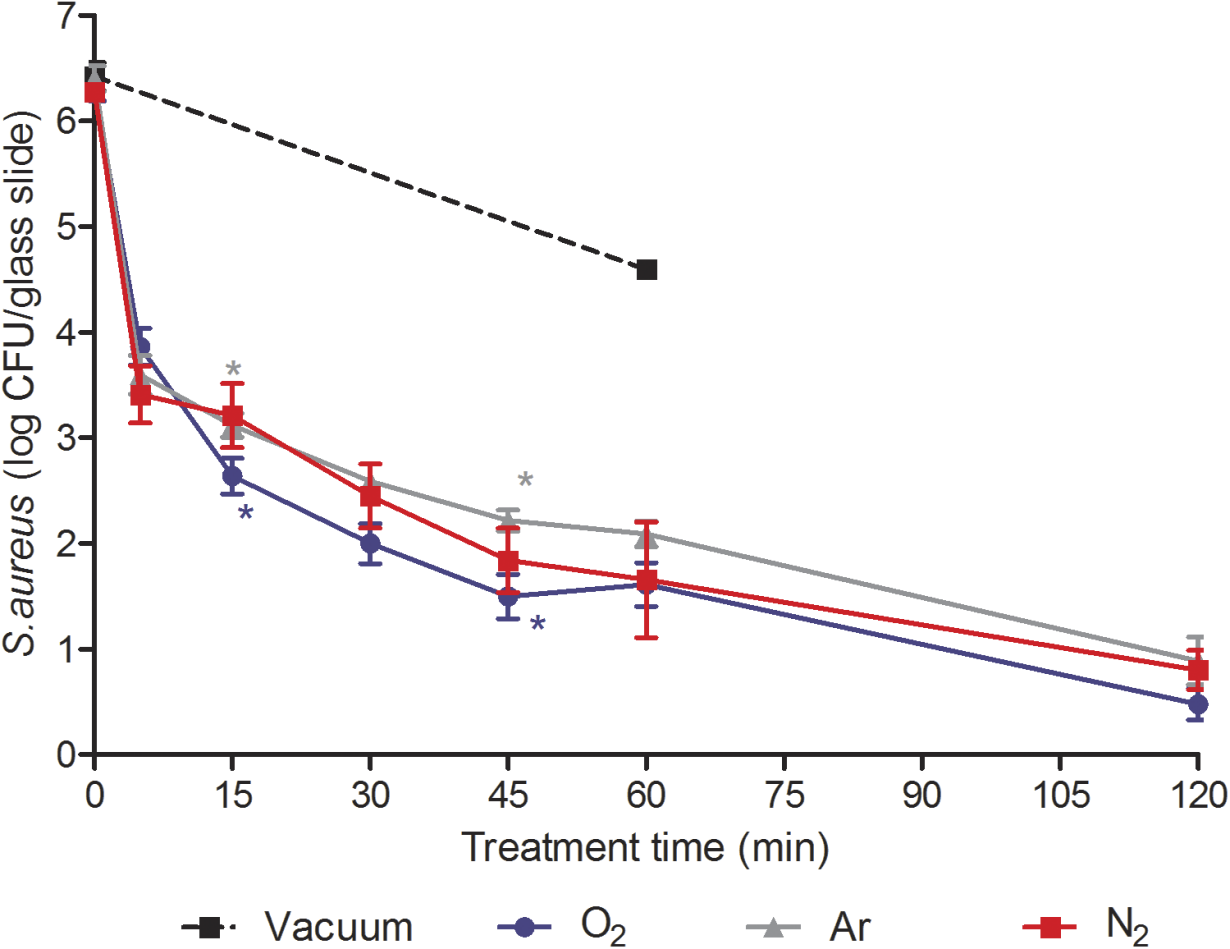
Survival curves of *S*. *aureus* exposed to N_2_, O_2_, Ar non-thermal plasma (25 W; 14 G and 0.5 sccm). Each data represents the mean of data obtained from at least six independent experiments made in triplicate. Statistical analysis was performed by the Student's t test (*p<0.05).

The bactericidal effect of plasma treatment was evaluated on *P*. *aeruginosa* (Fig 2) and *S*. *aureus* (Fig 3) with optimized prototype’s parameters (gas flow rate: 0.5 sccm; RF: 25 W; magnetic coils: 14 G), considering the potential impact of low pressure and temperature inside the bag on bacteria viability.

#### Low pressure and temperature effect on *P*. *aeruginosa* and *S*. *aureus*

During the process of sterilization, the samples were subjected to low pressure prior to plasma treatment. To identify the low pressure effect from plasma effect, some contaminated samples were subjected only to the low pressure without injection of gas for a 45-min period for *P*. *aeruginosa* and a 60-min period for *S*. *aureus*. At the end of the treatments, the vacuum level decreased up to 8.10^−5^ mbar in 45 min and 10^−5^ mbar in 60 min. The results showed a 1.8 log reduction of *P*. *aeruginosa* viability (Fig 2) and a 1.73 log reduction of *S*. *aureus* viability (Fig 3) compared to viability controls respectively. The direct effect of low pressure as well as temperature (< 40°C) could not solely be responsible for the bactericidal effect on *P*. *aeruginosa* and *S*. *aureus*. Moreover, when only low pressure was applied, the vacuum level decreased up to 8.10^−5^ mbar or 10^−5^ mbar compared to a plasma treatment (1.45. 10^−4^ mbar) in 45 or 120 min respectively. As a consequence, the results of the impact of low pressure on bacteria were probably overstated.

#### Plasma effect on *P*. *aeruginosa* viability

Regardless of the gas type, a time-dependent inactivation of *P*. *aeruginosa* inside the bags was obtained within a 45-min plasma treatment (Fig 2). As shown in Fig 2, the response of *P*. *aeruginosa* survival to plasma exposure time suggested a biphasic inactivation. A first fast reduction of bacterial viability (between 3.4 and 4.3 log CFU/glass slide) was observed within 5 min followed by a slower reduction up to 45 min. After a 5-min exposure, nitrogen plasma tended to be more effective than argon plasma (p < 0.01). However a prolonged treatment up to 45 min showed a similar effectiveness of the three treatments (p > 0.05) on the bacterial viability. After a 45-min exposure, we observed a 6 log reduction with O_2_, N_2_ and Ar plasma.

#### Plasma effect on *S*. *aureus* viability

Similarly, whatever the gas type, biphasic curves were observed with a fast reduction (between 2.5 and 2.9 log CFU/glass slide) of bacterial viability within 5 min followed by a slower reduction up to 120 min (Fig 3). The O_2_ plasma treatment seemed to be more efficient than Ar treatment after a 15-min and 45-min exposure. However, a 6 log reduction (p > 0.05) of bacteria viability was obtained after a 120-min treatment (p > 0.05) whatever the gas used.

### Effectiveness of plasma treatment on *B*. *subtilis* spores

#### Optimization of the discharge operating condition to inactive bacteria spores

Similar parameters to those used for the bacteria (gas flow rate: 0.5 sccm, RF: 25W) were applied for spores but we observed only 1 log reduction of spore viability after a 120-min treatment whatever the gas used (data not shown). Thus, to inactivate efficiently these highly resistant microorganisms, the operating conditions of the plasma had to be optimized. In our discharge operating conditions the flow rate of the gas(es) and the applied RF power could be modified while preserving the integrity of the sterilization bag. Indeed, the results showed with a gas flow rate up to 1 sccm that the applied RF power can increase up to 100W with O_2_, 75W with Ar and only 25W with N_2_ for a 120-min treatment. Beyond these RF power values, yellowing of the bags appeared.

#### Low pressure and temperature effect on *B*. *subtilis* spores

Similarly to bacteria, contaminated glass slides were subjected only to the low pressure without injection of gas for a 120 min period. The results showed no reduction of *spore viability* (data not shown). Thus the effect of low pressure as well as temperature (< 40°C) could not solely be responsible for the sporicidal effect on *B*. *subtilis*.

#### Plasma effect on *B*. *subtilis* viability

**Fig 4:**
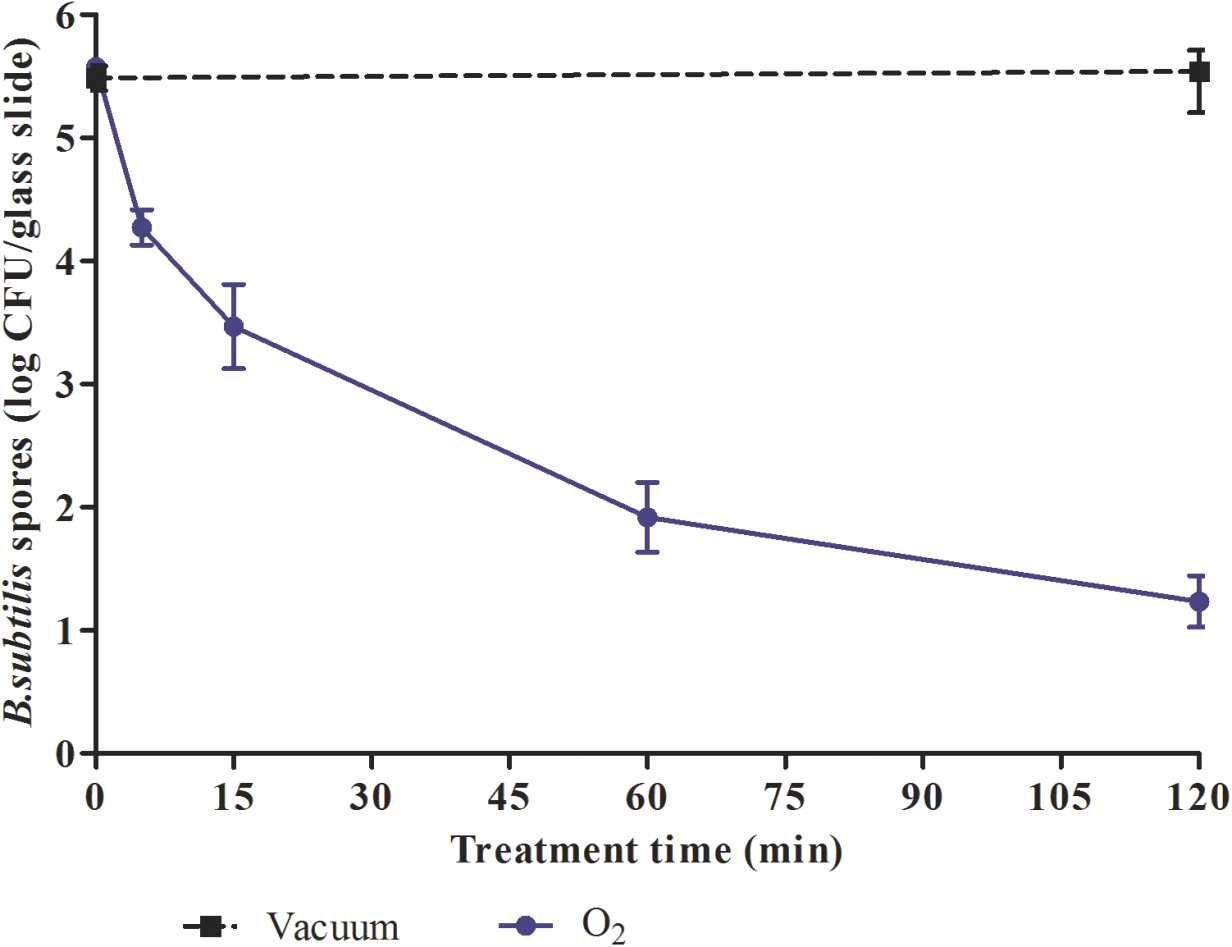
Survival curves of *B*. *subtilis* spores exposed to O_2_ non-thermal plasma (100 W; 14 G and 1 sccm). Each data represents the mean of data obtained from at least six independent experiments made in triplicate.

N_2_ and Ar treatments led only to a 2.43 log and 2.78 log reduction respectively after a 120-min treatment (data not shown). Only O_2_ treatment of 120 min led to a 4.3 log reduction of spore viability (Fig 4). No biphasic curve could be observed.

### Plasma effect on bacteria and spores morphology

#### Plasma effect on *P*.*aeruginosa* morphology

**Fig 5:**
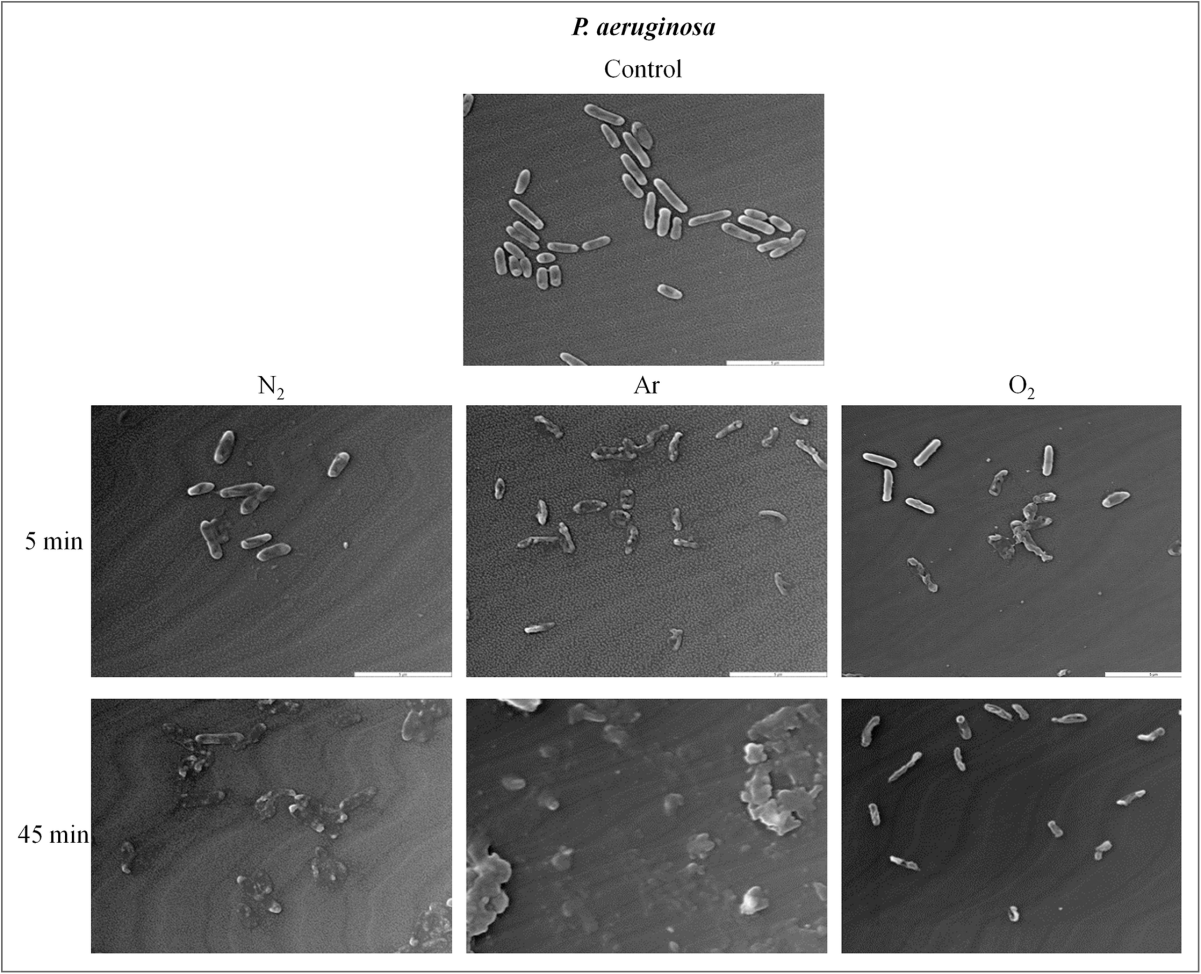
Scanning electron micrograph of *P*. *aeruginosa* before and after plasma treatments (25 W; 14 G and 0.5 sccm), (G x 7,500) (Jeol JSM 5400LV). (A) Untreated. (B, E) After N_2_ plasma treatment. (C, F) After Ar plasma treatment. (D, G) After O_2_ plasma treatment.

As seen by SEM, *P*. *aeruginosa* controls showed an ellipsoidal shape characteristic (Fig 5A). After plasma treatments (N_2_, Ar, O_2_), *P*. *aeruginosa* presented severe morphological alterations. After 5 min of plasma exposure, numerous holes were detected in cell walls (Fig 5B, 5C and 5D). After a 45-min treatment Ar and N_2_ plasma exposure seemed to induce more damaged than O_2_. Only some bacterial debris could be seen (Fig 5E and 5F) with both gases as O_2_ exposure led to ruptures in cell membranes but the bacteria shape could still be observed (Fig 5G).

#### Plasma effect on *S*.*aureus* morphology

**Fig 6:**
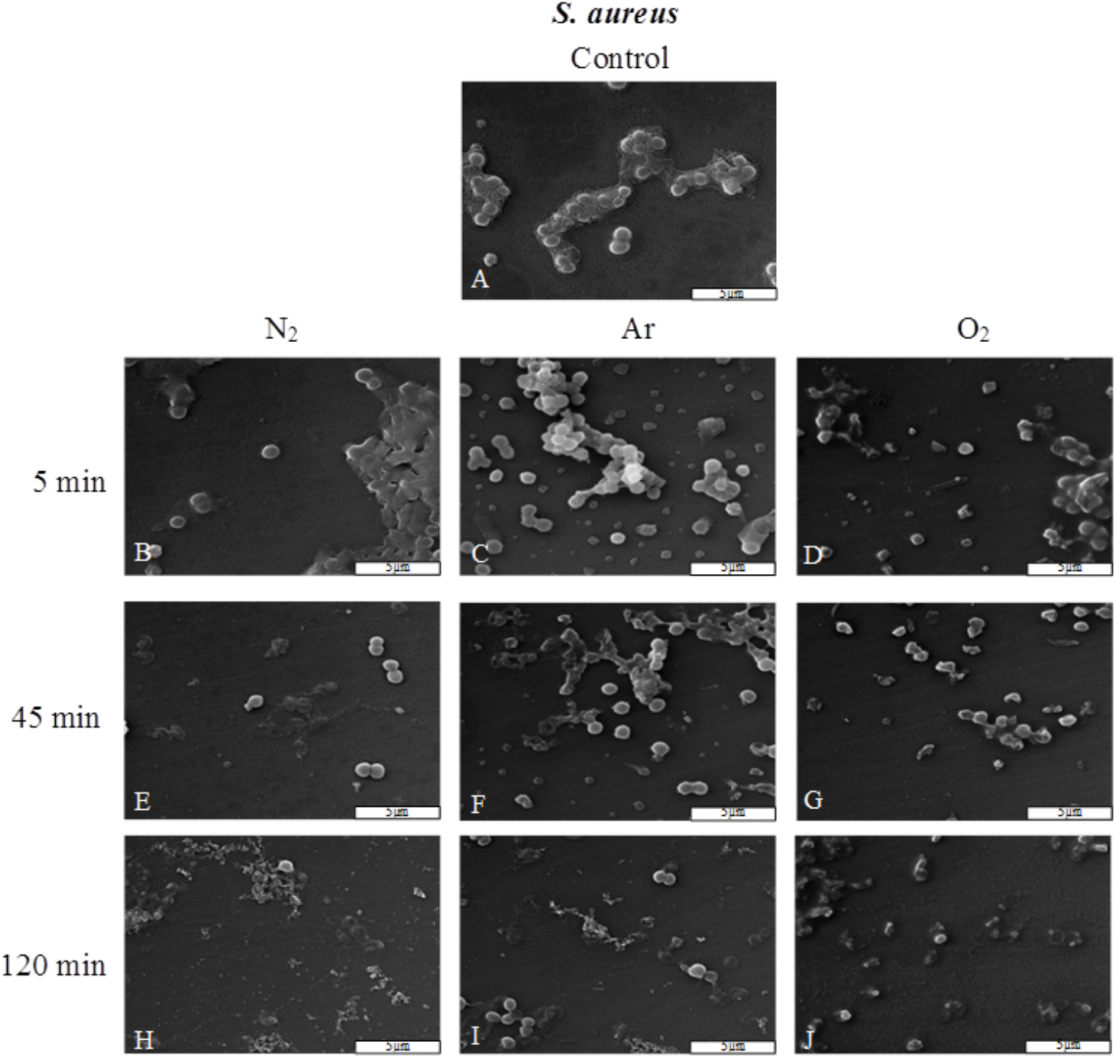
Scanning electron micrograph of *S*. *aureus* before and after plasma treatments (25 W; 14 G and 0.5 sccm), (G x 7,500). (A) Untreated. (B, E, H) After N_2_ plasma treatment. (C, F, I). After Ar plasma treatment. (D, G, J). After O_2_ plasma treatment.

The observation by SEM showed that *S*. *aureus* controls had a round shape and were covered by some exopolymers (Fig 6A). After 5 min of N_2_, Ar, O_2_ plasma exposure, only some bacteria were altered (Fig 6B, 6C and 6D) and the exopolymers seemed to be vitrified (Fig 6B). At the end of the treatment (120-min plasma exposure) there was a lot of bacterial debris and only a few bacteria could be visualized (Fig 6H, 6I and 6J).

#### Plasma effect on spore morphology

**Fig 7:**
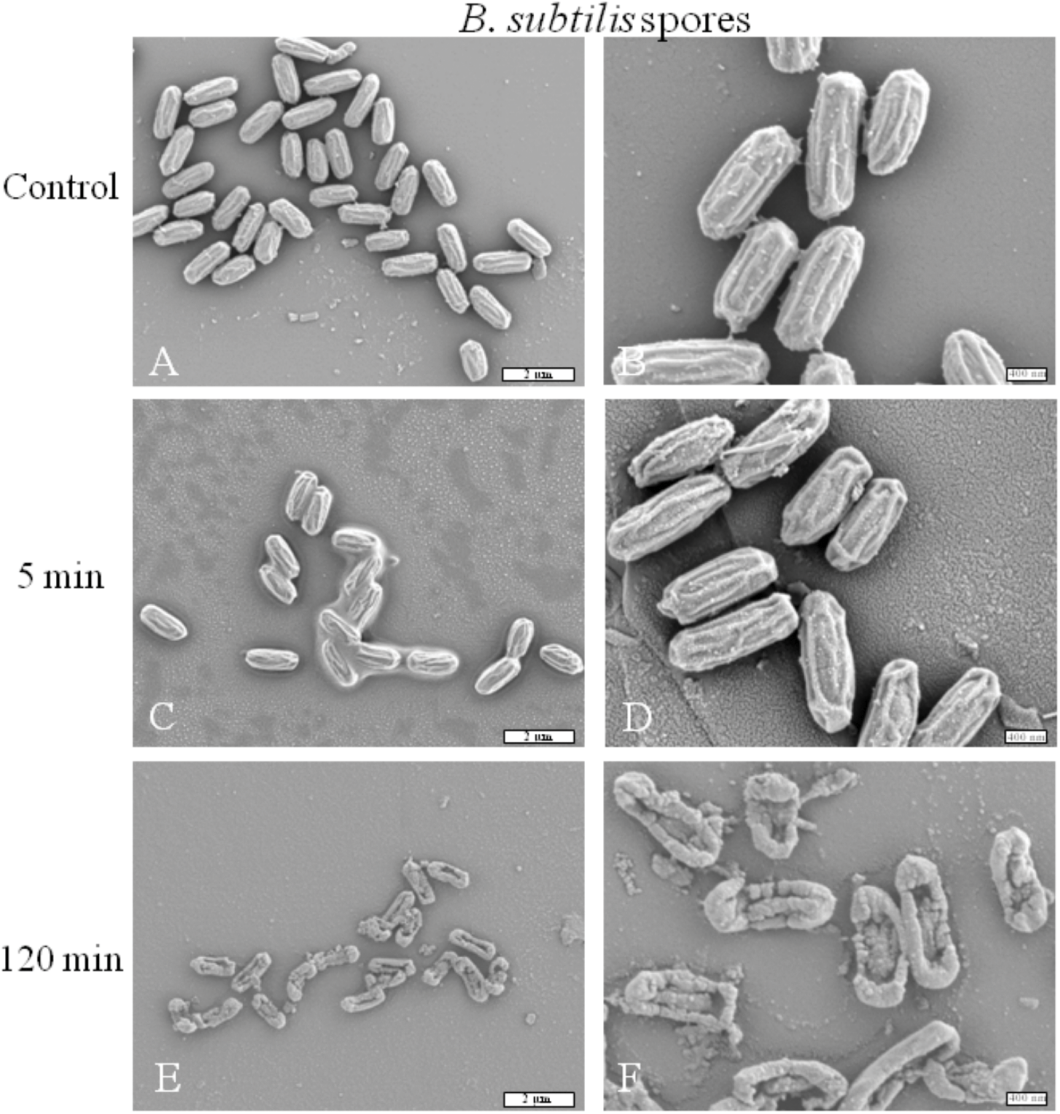
Scanning electron micrograph of *B*. *subtilis* spores before and after O_2_ gas plasma treatments (100 W; 14 G and 1 sccm), (G x 7,500 for the left column and G x 20,0000 for the right column). (A-B) Untreated. (C-F) After O_2_ plasma treatment.

SEM was used to examine the morphologic and structural changes of *B*. *subtilis* spores after plasma exposure. These changes are shown in Fig 7. After a 5-min treatment (Fig 7C and 7D), no visualized damages could be observed compared to the controls. (Fig 7A and 7B). After a 120-min O_2_ treatment (Fig 7E and 7F), only debris and some altered spores could be observed. These spores showed severe physical damages on their surface. The degradation of these cells is particularly visible at the central part of their body with the presence of holes through the layers whereas extremities are always visible.

### Bag integrity: FTIR and XPS analyses

During plasma treatments no bag perforation was noticed for the low density polyethylene bags. This continuous treatment could be demonstrated by the fact that the low pressure has never been broken during treatment process conditions, as low pressure is a necessary parameter for maintaining the plasma.

**Fig 8:**
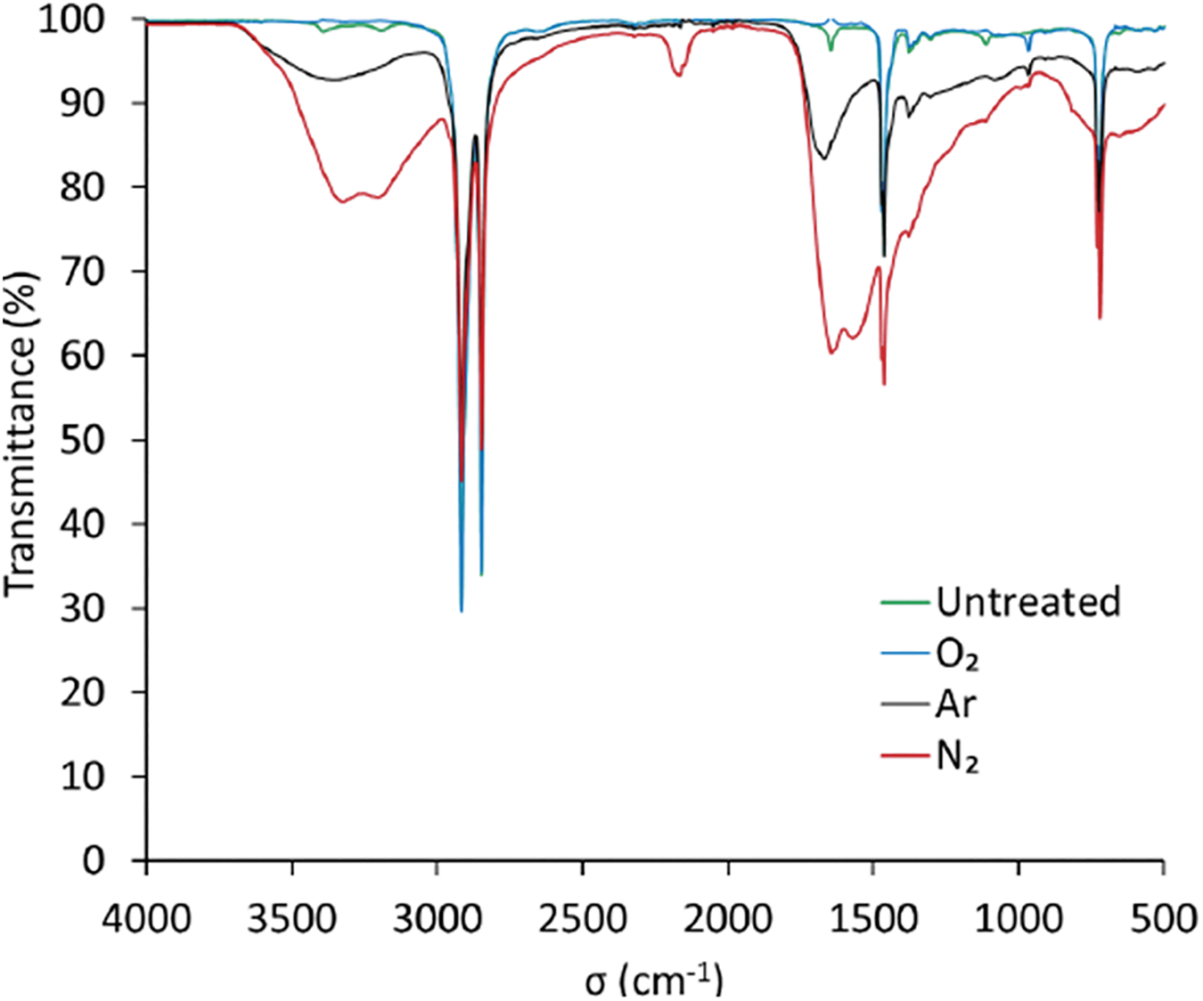
ATR-FTIR spectra of the PE film for untreated and 120 min plasma treatment at 100 W of O_2_, Ar and N_2_.

FTIR analysis showed some bands between 3000–3500 cm^-1^ and 800–1500 cm^-1^ and 800–1500 cm^-1^ for the untreated film (Fig 8). These bands are not necessarily attributed to the polyethylene but are probably derived from the additives during manufacturing.

Contrary to O_2_ plasma, after Ar and N_2_ treatments with 100 W RF power, a yellowing of the bags has been observed, which could be problematic, especially during prolonged storage, which could lead to an accelerated aging. For these conditions, the nitrogen plasma exhibits the creation of new macromolecular chains with four bands around 3340, 3140, 2200 and 1600 cm^-1^, some of which are assignable to amine groups (Fig 8). For treatment with argon plasma, practically the same bands appear with less intensity, attesting a functionalization by rupture of lateral bonds or chain bonds allowing a post-treatment reaction with nitrogen of the ambient air and then leading to grafting of the same amine groups.

**Fig 9:**
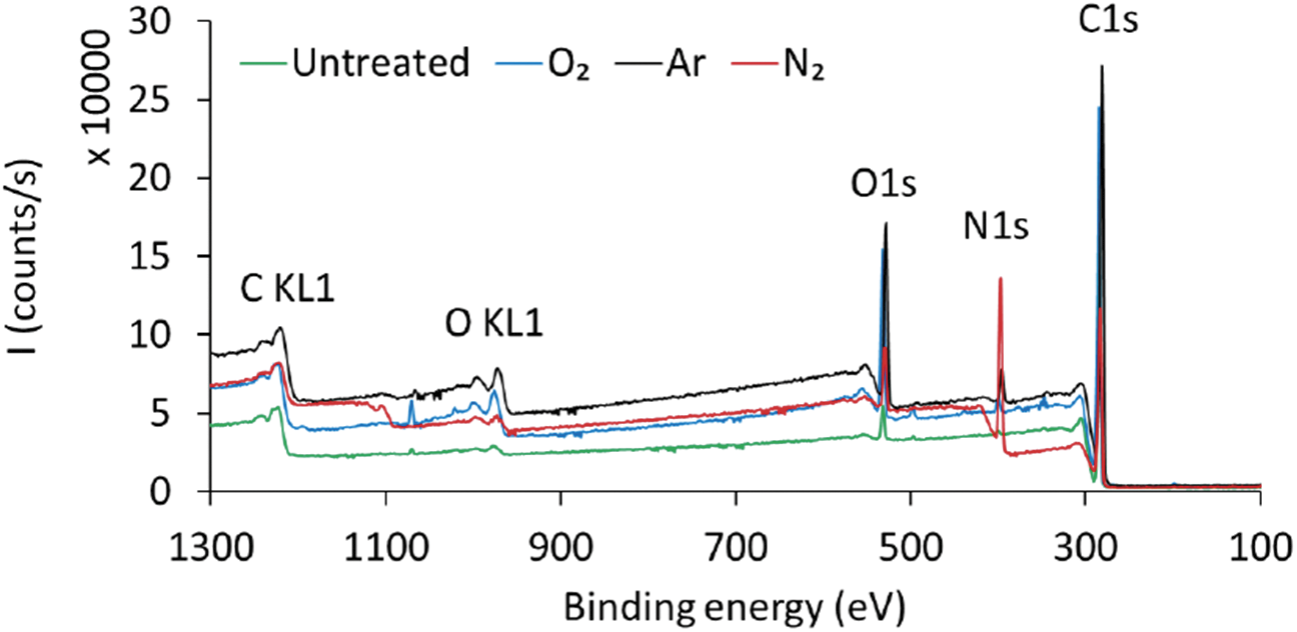
XPS survey scans of the PE bag film surfaces for untreated and 120 min plasma treatment at 100 W with O_2_, Ar and N_2_.

Compared with FTIR technique, XPS is a more accurate and sensitive technique to analyze surface chemical structure. Fig 9 presents the XPS survey scan spectra for the untreated and 120-min O_2_, Ar and N_2_ plasma treatments with 100 W RF power. The photoelectron peaks at binding energies 285 eV, 532 eV, and 400 eV corresponds to the C1s, O1s and N1s orbits, respectively. The elemental content of C decreased whereas the elemental contents of O and N increased for all the plasma treatment groups.

**Fig 10:**
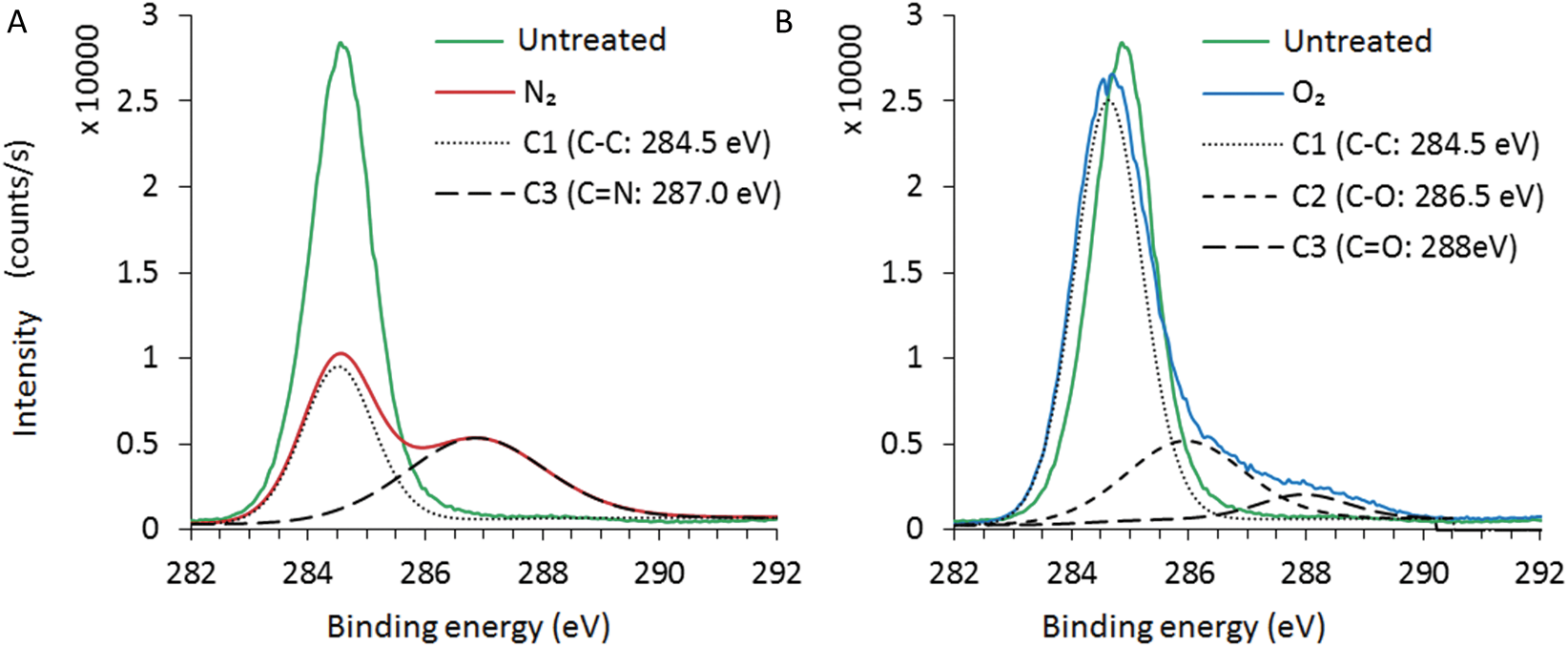
Comparison of the deconvolution of the XPS core level C1s spectra for untreated and 120 min plasma treatment at 100 W with N_2_ and O_2_. (A) Control. (B) Nitrogen.

In order to examine the changes in chemical functional groups after plasma treatment, the high resolution Cls spectrum was calculated by deconvolution and the detailed data are shown in Fig 10. Based on the literatures the C1s spectra of the control and nitrogen treated bags can be decomposed into two contributions appearing at 284.5 eV and 287.0 eV, which are assigned to the C-C and C = N, groups, respectively [http://www.lasurface.com/xps/index.php]. On the other hand, in the case of oxygen plasma treatment, the deconvolution did not result to any amine group. This is in good agreement with the FTIR analysis, which substantiates the creation of some new amine groups in the case of nitrogen or argon treatments that could be related to the yellowing apparition after the treatments at 100 W.

## Discussion

Sterilization processes have to be in accordance with aseptic methods (NF EN 556) allowing the delivery of enclosed products to the end-user. To reach this aim, a suitable, safe, and effective packaging maintaining the sterile state of items after the end of the sterilization process is a crucial factor. Despite numerous plasma sterilization processes, none were able, up to now, to preserve the sterile state after the treatment with regards to international standards.

To overcome this problem, our process generated a non-thermal plasma (θ ≥ 40°C) directly and solely inside a sealed bag without its deterioration. Three plasmas based on N_2_, Ar or O_2_ gas were tested as: 1) they are well-known for their antibacterial and sporicidal effect [6, 9, 11, 13, 16, 29]; 2) they do not require venting time at the end of the sterilization process like with ethylene oxide or ozone processes; 3) they are non-toxic [6].

The antimicrobial effects of non-thermal plasma generated in the sealed bags were tested on two vegetative bacteria, one Gram negative -*P*. *aeruginosa*-, and one Gram positive -*S*. *aureus*- and on *B*. *subtilis* spores, considered as sterilization bioindicators according to the NF EN 556 Norm. In our conditions, the temperature not exceeding 40°C and the low pressure did not induce a significant cell viability reduction. These antimicrobial effects could be explained by both the difference of the composition (membranes/layers) of microorganisms and the different inactivation mechanisms involved in plasma treatment as discussed in the following.

Gram negative and Gram positive bacteria cell wall are different although both have a protective role for cytoplasm. For example, Gram-negative bacteria have an additional lipid bilayer. The outer membrane presents phospholipids, lipopolysaccharides, lipoproteins [30–31]. The phospholipids are easily peroxidised during oxidative stress, such as during plasma treatment, compromising the structural integrity of the membrane [7, 31–35]. Gram-negative and Gram-positive bacteria have also different structure and nature of peptidoglycans [30]. Indeed the peptidoglycan of Gram-positive bacteria (30–100 nm thick) is thicker than Gram-negative bacteria (10–15 nm) [30] and it presents a high level of cross-linking due to the presence of numerous peptides chains between the alternating N-acetylglucosamine (NAG)-N-acetylmuramic disaccharide (NAM) units [30, 36]. Gram-positive bacteria like *S*. *aureus* have a peptidoglycan with 74 to 92% of cross-linkage whereas Gram-negative bacteria like *P*. *aeruginosa* have 35% of cross-linkage [36–37].

Plasma species need more time to alter Gram positive than Gram negative bacteria due to their differences of membranes such as thickness, structure and nature of peptidoglycan layer. Consequently, UV penetration is limited, and DNA destruction is delayed. Indeed, we obtained a 6 log reduction of *P*. *aeruginosa* and *S*. *aureus* viability in 45 min and 120 min respectively with our conditions: low pressure (1.45. 10^−4^ mbar), RF power (25 W), gas flow rate (0.5 sccm) and magnetic coils (14 G). These differences in inactivation efficiency between the two species are indeed correlated with bacterial morphology analyses by SEM. *P*. *aeruginosa* and *S*. *aureus* exposed to plasma treatments are damaged. But it appears clearly that Gram-positive *S*. *aureus* are more resistant to N_2_, Ar and O_2_ plasma than Gram-negative *P*. *aeruginosa*.

Spores present a complex structure composed of several layers (coat, outer membrane, cortex, inner membrane and core) conferring a high level of resistance compared to vegetative bacteria. Indeed, the spore coat of *B*. *subtilis* is a complex structure described as a ‘reactive shield’ to prevent access to plasma species to the inner [14]. These multilayers enhance resistance to plasma treatment making it an excellent sterilization bioindicator. Regarding the plasma treatment on *B*. *subtilis* spores, we had to increase the RF power and the gas flow rate to satisfy European Norms. Indeed we obtained a 4 log reduction in a 120-min treatment only with an O_2_ plasma with a gas flow rate (1 sccm) and a RF power (100 W). According to Bol’shakov *et al*., increasing RF power with a higher gas flow rate may induce higher electrons and ions density leading to a better destruction of spores [38]. After a 120-min O_2_ plasma treatment, SEM preparations are in correlation with the plasma effects as they showed that several spores were totally ruptured, jeopardizing the spore viability and a potential germination. To reduce the plasma exposure time, which is quite rather long (120 min), we currently investigate the performance of our equipment on *B*. *subtilis* spores, with different gases mixtures in combination with an increase of gas flow rate, while preserving the bag integrity.

Throughout the plasma treatment, different mechanisms occur to inactive microorganisms, involving neutral, ionized and reactive species as well as UV photons [5, 7]. With our plasma conditions of sterilization the bactericidal curves of both bacteria strains were biphasic. Similarly numerous studies dealing with plasma sterilization generated at atmospheric, medium or low pressure have shown the same results [6, 8, 14, 33, 39]. However different curve profiles, such as single-slope curve or triphasic curve can be observed in other studies [7, 13, 21, 40–42]. Taking into consideration the current literature, different factors influence the efficiency of plasma which are inherent to microorganisms (*e*.*g*. type of microorganisms—bacteria/spores—, number of layers—monolayer/multilayers -) and inherent to plasma processes (*e*.*g*. reactor set-up, operating pressure, power, nature and flow rate of the gas…) [6, 8, 26, 29,31, 33, 40, 41, 43–45]; thus it is likely that more than one mechanism be involved in plasma microorganism inactivation.

Consequently, the exact mechanisms of interactions between non-thermal plasmas and microorganisms remain to be considered but several authors suggest that the first bacterial inactivation phase, which exhibit the fastest decrease, is dominated by the UV radiations on isolated microorganisms [6–7, 21, 46]. Accumulated UV radiations damage the cell wall of bacteria, then they penetrate into the cell to damage DNA strands *via* a dimerization of the thymine base, thus inhibiting the replication ability of the bacteria. Simultaneously, charged particles and RONS initiate the alteration of bacterial membranes by photodesorption or etching mechanisms [1, 6, 8]. However, spores are known to be very resistant to UV radiations [31, 47–49]. UV photons energy emitted in O_2_ plasma could be quickly dissipated through the various spore layers restricting UV photons actions at the outer layers [7, 50]. This limited penetration of these species could explain the difference of plasma efficiency between vegetative bacteria and spores.The second bacterial inactivation phase, with a lower decrease of bacteria inactivation might match the inactivation of under-layer bacteria which were shielded by other bacteria and covered by some debris. The presence of organic material limits the penetration of different plasma species by protecting the bacteria inducing a lower inactivation [43, 51]. This phase is dominated by an erosion process through photodesorption by UV photons or through etching by active species [5, 7, 10, 33]. During this phase, RONS attack the unsaturated fatty acids which are highly present in Gram negative cell membrane [31, 52]; oxygen atoms can also diffuse into the cell causing oxidative stress possibly leading to antimicrobial effect [53].

In a previous study [26], we have tested non-thermal plasma on *P*. *aeruginosa* multilayer biofilms *versus* monolayer biofilms and we showed that a 5% O_2_−95% N_2_ non-thermal plasma treatment of 30 min led to a 5 log (CFU/mm^2^) reduction for a multilayer biofilm and a 6 log reduction for a monolayer biofilm. This highlights the fact that the presence of organic material reduces the penetration of plasma species through the layers. But we also showed that extending the duration treatment increased the plasma efficiency on multilayer biofilms. Furthermore, this plasma was able to reduce *P*. *aeruginosa* biofilms cultured on titanium alloy coated with hydroxyapatite presenting some roughness and porosities.

Although no biphasic curve was observed during *B*. *subtilis* spores treatment, spores are totally damaged after a 120-min O_2_ plasma treatment. Our SEM observations showed an alteration of all layers of the spores suggesting an inability to germinate. The efficient inactivation of spores in oxygen-based plasmas has been already reported at low pressure plasma discharges [46]. Plasma exposures for longer periods would often lead to pan-oxidation of the cells, leading to oxidative damage. Thus, mainly reactive plasma species react with almost all cell components including membranes, DNA, proteins leading to damage affecting their germination process [14, 24, 31, 44].

The antimicrobial effects of our non-thermal plasmas have been successfully demonstrated on different microorganisms. One other aspect is the bag integrity that must be also preserved under high vacuum in order to maintain the sterile state of single use items. To examine potential low density polyethylene modifications of bags, FTIR and XPS analysis were performed downstream the plasma treatment with the selected parameters for spores inactivation, considered as the worst-case process. Thus, for the treatment conditions of 100 W, no amine group was detected for O_2_ plasma treatment which could correlated with no modification of the bag color whereas with N_2_ and Ar plasmas amine groups have been detected and could have resulted in the yellowing/browning of the bags. Sarrette *et al*. [22] have tested different membranes made of polypropylene or cellulose with a N_2_ microwave flowing afterglow set-up. They showed that there was no significant N-atoms losses during the crossing of polypropylene membranes. This type of membrane could be an opportunity to be used as a bag of sterilization but it will be necessary to look more closely to the problematic of polymer bag behavior during the different plasma processes.

Moreover, the long-term integrity of the bags is a critical point for the sterile state preservation of items. As the just-in-time production is currently preferred as an economic model, the objective is to preserve the sterile state for a limited time. Some bactericidal tests and physical tests on bags are in progress. Thus considering the bacteriological results and the bag characterization the process parameters for further studies will be an O_2_ plasma a 120-min exposure time, with 1 sccm gas flow rate, a 100W RF power and 14 G magnetic coils.

## Conclusions

Preserving the bag integrity downstream of the process is a major point to maintain the sterile state of the sterilized device. The innovative technique described here represents therefore an important step forward in the field of plasma sterilization applications in accordance with the NF EN 556 Norm. This technique, less aggressive for the heat-sensitive medical devices and avoiding toxic risks for the operators, can be considered as a solution for the sterilization of single use medical devices that do not so far support conventional sterilization methods.
